# Deprivation and Health Care Access and Utilization of Participants with Glaucoma in the NIH All of Us Research Program

**DOI:** 10.51894/001c.164372

**Published:** 2026-07-31

**Authors:** Ambika Salwan, Patrice M. Hicks, Jacob Goldstein-Greenwood, Joshua R. Ehrlich, Maria A. Woodward, Angela R. Elam, David B. Rein, David C. Musch, Roshanak Mehdipanah, Jayant M. Pinto, Sung Kyun Park, Donglin Zeng, Paula Anne Newman-Casey

**Affiliations:** 1 The University of Michigan

## 38

### Purpose

To assess if community deprivation is associated with health care access and utilization in participants in the NIH All of Us Research Program with glaucoma.

### Methods

We analyzed associations between three-digit ZIP Code (ZIP3) area-level deprivation and 15 person-level binary outcomes capturing the accessibility and use of health care resources (e.g., delaying care due to the copay) in US glaucoma patients diagnosed at ≥ 40 years of age. We used logistic generalized estimating equations with a logit link function to account for clustering of binary outcomes within ZIP3. We fit adjusted models that included age, sex, race/ethnicity, and education, and models that further controlled for individual-level household income bracket, employment status, and housing arrangement.

### Results

Data for 5881 people across 379 ZIP3s were usable in ≥1 model (median age: 71; 55% female; 69% white; 58% at least college educated). When adjusting for age, sex, race/ethnicity, and education level, deprivation index (rescaled [0, 10]) was associated with being unable to afford eyeglasses (OR [95% CI]: 1.21 [1.04, 1.40]; *p* = .02), lacking follow-up (OR [95% CI]: 1.32 [1.08, 1.61]; *p* = .01) or emergency (OR [95% CI]: 1.35 [1.03, 1.77]; *p* = .03) care, and receiving delayed care due to the copay (OR [95% CI]: 1.36 [1.02, 1.83]; *p* = .04). Further adjustment for household income, employment status, and housing arrangement, deprivation index was not significantly associated with any outcomes.

### Conclusion

In US glaucoma patients diagnosed at ≥ 40 years of age, ZIP3-level deprivation was linked to greater odds of cost-related care barriers after adjusting for age, sex, race/ethnicity, and education, but these associations appear to be explained by individual-level income, employment, and housing. Future research should assess the mechanisms that explain these relationships toward improved glaucoma access and clinical outcomes.

